# High-flow nasal oxygen in patients with COVID-19-associated acute respiratory failure

**DOI:** 10.1186/s13054-021-03469-w

**Published:** 2021-02-11

**Authors:** Ricard Mellado-Artigas, Bruno L. Ferreyro, Federico Angriman, María Hernández-Sanz, Egoitz Arruti, Antoni Torres, Jesús Villar, Laurent Brochard, Carlos Ferrando, for the COVID-19 Spanish ICU Network, Ricard Mellado-Artigas, María Hernández-Sanz, Carlos Ferrando, Marina Vendrell, Gerard Sánchez-Etayo, Amalia Alcón, Isabel Belda, Mercé Agustí, Albert Carramiñana, Isabel Gracia, Miriam Panzeri, Irene León, Jaume Balust, Ricard Navarro, María José Arguís, María José Carretero, Cristina Ibáñez, Juan Perdomo, Antonio López, Manuel López-Baamonde, Tomás Cuñat, Marta Ubré, Antonio Ojeda, Andrea Calvo, Eva Rivas, Paola Hurtado, Roger Pujol, Nuria Martín, Javier Tercero, Pepe Sanahuja, Marta Magaldi, Miquel Coca, Elena del Rio, Julia Martínez-Ocon, Paula Masgoret, Monserrat Tio, Angel Caballero, Raquel Risco, Raquel Bergé, Lidia Gómez, Nicolás de Riva, Ana Ruiz, Beatriz Tena, Sebastián Jaramillo, José María Balibrea, Francisco Borja de Borja de Lacy, Ana Otero, Ainitze Ibarzabal, Raquel Bravo, Anna Carreras, Daniel Martín-Barreda, Alfonso Jesús Alias, Mariano Balaguer, Jorge Aliaga, Alex Almuedo, Joan Ramón Alonso, Rut Andrea, Gerard Sergi Angelès, Marilyn Arias, Fátima Aziz, Joan Ramon Badía, Enric Barbeta, Toni Torres, Guillem Batiste, Pau Benet, Xavi Borrat, María Borrell, Ernest Bragulat, Inmaculada Carmona, Manuel Castellà, Pedro Castro, Joan Ceravalls, Oscar Comino, Claudia Cucciniello, Clàudia De Deray, Oriol De Diego, Paula De la Matta, Marta Farrero, Javier Fernández, Sara Fernández, Anna Fernández, Miquel Ferrer, Ana Fervienza, María Tallo Forga, Daniel Forné, Clàudia Galán, Andrea Gómez, Eduard Guasch, María Hernández-Tejero, Adriana Jacas, Beltrán Jiménez, Pere Leyes, Teresa López, José Antonio Martínez, Graciela Martínez-Pallí, Jordi Mercadal, Guido Muñoz, José Muñoz, Ricard Navarro, Josep María Nicolás, José Tomás Ortiz, Anna Peiró, Manuel Pérez, Esteban Poch, Margarida Pujol, Eduard Quintana, Bartomeu Ramis, Enric Reverter, Irene Rovira, Pablo Ruiz, Elena Sandoval, Stefan Schneider, Oriol Sibila, Carla Solé, Alex Soriano, Dolors Soy, M. Suárez, Adrián Téllez, Néstor David Toapanta, Antoni Torres, Xavier Urra, César Aldecoa, Alicia Bordell, Silvia Martín, Judith Andrés, Alberto Martínez Ruiz, Gonzalo Tamayo Medel, Iñaki Bilbao Bilbao Villasante, Fernando Iturri Clavero, Covadonga Peralta Álvarez, Julia T. Herrera Díez, Andrea García Trancho, Iñaki Sainz Mandiola, Carmen Ruano Suarez, Angela Ruiz Bocos, Eneritz Urrutia Izagirre, Pablo Ortiz de Urbina Fernández, Naiara Apodaka López, Leire Prieto Molano, Eunate Ganuza Martínez, Iratxe Vallinas Hidalgo, Karmele de Orte Sancho, Celia González Paniagua, Gemma Ortiz Labrador, Mireia Pérez Larrañaga, Marta López Miguelez, Estíbaliz Bárcena Andrés, Erik Urutxurtu Laureano, Maria Jesús Maroño Boedo, Blanca Escontrela Rodríguez, Aitziber Ereñozaga Camiruaga, Deiene Lasuen Aguirre, Ainhoa Zabal Maeztu, Ane Guereca Gala, Iker Castelo Korro, Andrés Álvarez Campo, Alejandro Carcelen Viana, Alejandro Alberdi Enríquez, Xabier Ormazábal Rementeria, Alberto Sánchez Campos, Rosa Gutiérrez Rico, Pablo Barbier Damborenea, Marta Guerenabarrena Momeñe, Borja Cuesta Ruiz, Alejandro López Rico, Ana Rojo Polo, Covadonga García Grijelmo, Mikel Celorrio Reta, Eneko Martín Arroyo, Leire Artaza Aparicio, Iñaki Ituarte Aspiazu, Ane Igeregi Basabe, Itxaso Merino Julian, Isabel Diaz Rico, Maria Paz Martínez, Ramón Adalia Bartolomé, Luigi Zattera, Irina Adalid Hernandez, Leire Larrañaga Altuna, Aina Serrallonga Castells, Adriana Vílchez Garcia, María Núñez, Lorena Román, Isabel Ramos Delgado, Adela Benítez-Cano Martínez, Mireia Chanzá Albert, Juan Carlos Álvarez García, Luis Aguilera Cuchillo, Sandra Beltrán de Heredia, Jesús Carazo Cordobés, Carlos Alberto García Bernedo, Fernando Escolano Villén, Francisco Javier Redondo Calvo, Rubén Villazala González, Victor Baladron González, Patricia Faba, Omar Montenegro, Natalia Bejarano Ramírez, Sergio Marcos Contreras, Alejandro Garcia Rodríguez, Saleta Rey Vázquez, Cristina Garcia Pérez, Eva Higuera Miguelez, Irene Pérez Blanco, David García Rivera, Ane Martín de la Fuente, Marta Pardo, Vanessa Rodriguez, Unai Bengoetxea, Fernando Ramasco, Sheila Olga Santidrián Bernal, Alvar Santa Cruz Hernando, Antonio Planas Roca, Carlos Figueroa Yusta, Esther García Villabona, Carmen Vallejo Lantero, Eva Patiño Rodriguez, Alvaro Esquivel Toledo, David Arribas Méndez, Mar Orts Rodriguez, Rosa Méndez Hernández, Jesús Nieves Alonso, Inés Imaz Artazcoz, Sonia Expósito Carazo, Carlos Román Guerrero, Elena Rojo Rodríguez, Ricardo Moreno González, Julia Hernando Santos, Jara Torrente Pérez, Esperanza Mata Mena, Manuel José Muñoz Martínez, Enrique Alday Muñoz, Patricia Martin Serrano, Laura Cotter Muñoz, Amadea Mjertan, Diego Gutierrez Martínez, Carmen Rodríguez García, Olaya Alonso Viejo, Juan Alvarez Pereira, Ana Carmona Bonet, Diana Parrado López, Eva de Dios Tomas, Rafael Martín Celemin, María Luisa Meilan Paz, Luis Quecedo Gutiérrez, Noemí Diaz Velasco, Gabriel Martin Hernández, Francisco Garcia del Corral, Gloria Hernandez Arias, David Rodriguez Cuesta, Ana Gómez Rice, Encarna Mateos Sevillano, Natalia Olmos Molpeceres, Beatriz Domínguez, Ana Vázquez Lima, Ángel Candela, Ismael A. Acevedo Bambaren, Maria Isabel Albala Blanco, Paloma Alonso Montoiro, Fernando Álvarez Utrera, Juan Avellanosa Esteruelas, Amal Azzam López, Alberto José Balvis, Balvis Tommaso Bardi, María Beltrán Martín, Jacobo Benatar Haserfaty, Alberto Berruezo Camacho, Laura Betolaza Weimer, María del Mar Carbonell Soto, Cristina Carrasco Seral, Cristina Cerro Zaballos, Elizabeth Claros Llamas, Pilar Coleta Orduna, Ingrid P. Cortes Forero, Pascual Agustín Crespo Aliseda, María Angélica de Pablo Pajares, Yolanda Díez Remesal, Trinidad Dorado Díaz, Noemí Echevarría Blasco, María Elena Elías Martín, Javier Felices Triviño, Natalia Fernández López, Cristina Fernández Martín, Natalia Ferreiro Pozuelo, Luis Gajate Martín, Clara Gallego Santos, Diego Gil Mayo, María Gómez Rojo, Claudia González Cibrián, Elena Herrera López, Borja Hinojal Olmedillo, Berta Iglesias Gallego, Sassan Khonsari, María Nuria Mane Ruiz, María Manzanero Arroyo, Ana María Mariscal Ortega, Sara Martín Burcio, María del Carmen Martín González, Ascensión Martín Grande, Jose Juan Martín López, Cecilia Martín Rabes, Marcos Martínez Borja, Nilda Martínez Castro, Adolfo Martínez Pérez, Snejana Matcan, Cristina Medrano Viñas, Lisset Miguel Herrera, Adrián Mira Betancur, María Montiel Carbajo, Javier Moya Moradas, Lorena Muñoz Pérez, Mónica Nuñez Murias, Eva Ordiales González, Óscar Ordoñez Recio, Miguel Ángel Palomero Rodriguez, Diego Parise Roux, Lucia Pereira Torres, David Pestaña Lagunas, Juana María Pinto Corraliza, Marian Prieto Rodrigo, Inmaculada Rodriguez Diaz-Regaño, David Rodriguez Esteban, Víctor Rojas Pernia, Álvaro Ruigómez Saiz, Bárbara Saavedra Villarino, Noemí Samaranch Palero, Gloria Santos Pérez, Jaume Serna Pérez, Ana Belén Serrano Romero, Jesús Tercero López, Carlos Tiscar García, Marta de la Torre Concostrina, Eva María Ureta Mesa, Eva Velasco Olarte, Judith Villahoz Martínez, Raúl Villalaba Palacios, Gema Villanueva García, Cristina Vogel de Medeiros, Soraya Gholamian Ovejero, Marta Vicente Orgaz, Patricia Lloreda Herradon, Cristina Crespo Gómez, Tatiana Sarmiento-Trujillo, Noemí García Medina, María Martínez García, Carles Espinós Ramírez, Nabil Mouhaffel Rivero, Jose Antonio Bernia Gil, Sonsoles Martín, María Victoria Moral, Josefina Galán, Pilar Paniagua, Sergio Pérez, Albert Bainac, Ana Arias, Elsa Ramil, Jorge Escudero, Pablo Monedero, Carmen Cara, Andrea Lara, Elena Mendez Martínez, Jorge Mendoza, Íñigo Rubio Baines, Carmen Sala Trull, Pablo Montero López, Alfredo Gea, Alejandro Montero, Rocío Armero Ibañez, Juan Vicente Llau Pitarch, Fernando Rauer Alcóver, Cristina Álvarez Herreros, Cyntia Sánchez Martín, Lucía López Ocáriz Olmos, Marta Navas Moruno, Fernando García Montoto, M. F. Mirón Rodriguez, Laura Fuentes Coco, Cristina Hernández Gamito, Antonio Barba Orejudo, Luis Gerardo Smith Vielma, Yasmina González Marín Francisco de Borja Amador Penco, Marta Donoso Domínguez, Silvia Esquivel Ramírez, José Antonio Carbonell, Berta Monleón López, Sara Martínez-Castro, Gerardo Aguilar, María Gestal Pablo Casas, Angel Outeiro Rosato, Andrea Naveiro Pan, María Alonso Portela, Adrián García Romar, Eva Mosquera Rodríguez, Diego Ruanova Seijo, Pablo Rama Maceiras, Francisco Castro-Ceoane, Esther Moreno López, Sergio Gil, Julia Guillén Antón, Patricia García-Consuegra Tirado, Aurora Callau Calvo, Laura Forés Lisbona, María Carbonell Romero, Belén Albericio Gil, Laura Pradal Jarne, María Soria Lozano, Diego Loscos López, Andrea Patiño Abarca, Jordi Serrano, Javier Pérez-Asenjo Ángel Díez-Domínguez, Ion Zubizarreta, Jon Ramos, Iosu Fernández, Emilio Maseda Alejandro Suárez de la Rica, Javier Veganzones, Itziar Insausti Javier Sagra, Sofía Díaz Carrasco, Ana Montero Feijoo, Julio Yagüe, Ignacio Garutti Javier Hortal, Patricia Piñeiro, Matilde Piñeiro, Matilde Zaballos, Jamil cedeño, Pablo García-Olivares, Alberto Garriido, Jose Eugenioi Guerrero, Eva Bassas Parga, Carmen Deiros Garcia Elisenda Pujol Rosa, Ana Tejedor Navarro, Roser Font Gabernet, Maria José Bernat, Meritxell Serra Valls, Cristina Cobaleda Garcia-Bernalt, Jesus Fernanz Anton, Adriana Aponte Sierra, Lucia Gil Gomez, Olaia Guenaga Vaqueiro, Susana Hernandez Marin, Laura Pardo Pinzon, Sira Garcia Aranda Carlos Briones Orejuela, Edgar Cortes Sánchez, Alejandro Romero Fernández, Esther Fernández SanJosé, Patricia Iglesias Garsabal, Guillermo Isidro Lopez, Ana Vicol, Sara Espejo Malagon, María Sanabra Loewe, Laura Grau Torradeflo Lourdes Blanco Alcaide, Gloria Buenaventura Sanclemente, Pere Serra Pujol Gustavo Cuadros Mendoza, Miroslawa Konarska, Fedra Bachs Almenara Agnieszka Golska, Aleix Carmona Blesa, Arantxa Mas Serra, Javier Ripolles Melchor, Ana Nieto Moreno, Káteri Chao Novo, Sandra Gadín López, Elena Nieto Moreno, Bérénice Gutiérrez Tonal, Elena Lucena de Pablo, Barbara Algar Yañez, Beatriz Vázquez Rivero, Beatriz Nozal Mateo, Marina de Retes, Norma Aracil Escoda, Cristina Gallardo Mayo, Rosa Sanz González, Alicia Ruiz Escobar, Maria Laura Pelegrina López Marina Valenzuela Peña, David Stolle Dueñas, Ane Abad Motos, Alfredo Abad-Gurumeta, Ana Tirado Errazquin, Elena Sáez Ruiz, Nerea Gómez Pérez, Francisco de Borja Bau González, Cesar Morcillo Serra Jessica Souto Higueras, Rosario Vicente Raquel Ferrandis, Silvia Polo Martín, Azucena Pajares Moncho, Ignacio Moreno Puigdollers, Juan Pérez Artacho Cortés, Ana Moret Calvo, Ana Pi Peña, María Catalán Fernández, Marina Varela Pilar Díaz Parada, Raquel Rey Carlín, Sarra Barreiro Aragunde, María Isabel Forés Chiva, A. Javier Agulló, Antonio Pérez Ferrer, María Galiana, Antoni Margarit Válerie Mourre del Rio, Eva Heras Muxella, Anna Vidal

**Affiliations:** 1 Department of Anesthesiology and Critical Care, Institut D’investigació August Pi I Sunyer, Hospital Clínic, Villarroel 170, 08025 Barcelona, Spain; 2 Department of Medicine, Sinai Health System and University Health Network, Toronto, Canada; 3 Interdepartmental Division of Critical Care Medicine, University of Toronto, Toronto, Canada; 4 Department of Critical Care Medicine, Sunnybrook Health Sciences Centre, Toronto, Canada; 5 Department of Anesthesiology and Critical Care, Hospital de Cruces, Vizcaya, Spain; 6 Ubikare Technology, Vizcaya, Spain; 7 Department of Respirology, Hospital Clínic, Institut D’investigació August Pi i Sunyer, Barcelona, Spain; 8 CIBER de Enfermedades Respiratorias, Instituto de Salud Carlos III, Madrid, Spain; 9 CIBERESUCICOVID, Instituto de Salud Carlos III, Madrid, Spain; 10 Multidisciplinary Organ Dysfunction Evaluation Research Network, Research Unit, Hospital Universitario Dr. Negrin, Las Palmas de Gran Canaria, Spain; 11 Keenan Research Centre for Biomedical Science at the Li Kan Shing Knowledge Institute, St Michael’s Hospital, Toronto, ON Canada

**Keywords:** COVID-19, Acute hypoxemic respiratory failure, High-flow nasal oxygen, Ventilator-free days

## Abstract

**Purpose:**

Whether the use of high-flow nasal oxygen in adult patients with COVID-19 associated acute respiratory failure improves clinically relevant outcomes remains unclear. We thus sought to assess the effect of high-flow nasal oxygen on ventilator-free days, compared to early initiation of invasive mechanical ventilation, on adult patients with COVID-19.

**Methods:**

We conducted a multicentre cohort study using a prospectively collected database of patients with COVID-19 associated acute respiratory failure admitted to 36 Spanish and Andorran intensive care units (ICUs). Main exposure was the use of high-flow nasal oxygen (conservative group), while early invasive mechanical ventilation (within the first day of ICU admission; early intubation group) served as the comparator. The primary outcome was ventilator-free days at 28 days. ICU length of stay and all-cause in-hospital mortality served as secondary outcomes. We used propensity score matching to adjust for measured confounding.

**Results:**

Out of 468 eligible patients, a total of 122 matched patients were included in the present analysis (61 for each group). When compared to early intubation, the use of high-flow nasal oxygen was associated with an increase in ventilator-free days (mean difference: 8.0 days; 95% confidence interval (CI): 4.4 to 11.7 days) and a reduction in ICU length of stay (mean difference: − 8.2 days; 95% CI − 12.7 to − 3.6 days). No difference was observed in all-cause in-hospital mortality between groups (odds ratio: 0.64; 95% CI: 0.25 to 1.64).

**Conclusions:**

The use of high-flow nasal oxygen upon ICU admission in adult patients with COVID-19 related acute hypoxemic respiratory failure may lead to an increase in ventilator-free days and a reduction in ICU length of stay, when compared to early initiation of invasive mechanical ventilation. Future studies should confirm our findings.

## Introduction

High-flow nasal oxygen (HFNO) reduces the need for intubation in adult patients with acute respiratory failure [1–4]. This may in turn help to avoid the associated risks of invasive mechanical ventilation, such as delirium and cognitive impairment, intensive care unit (ICU) acquired weakness and secondary infections. However, through vigorous breathing efforts, spontaneous ventilation could theoretically promote further lung injury (e.g., patient self-inflicted lung injury) [5–9].

A novel coronavirus disease (COVID-19) has spread worldwide causing thousands of cases of acute respiratory failure with a high mortality rate [10, 11]. Thus far, the use of HFNO has been limited, despite the fact it may represent an appropriate initial therapy [12, 13]. Conversely, several studies have shown that the use of invasive mechanical ventilation remains high in this population, and patients usually receive it for prolonged periods of time [14–16]. In daily clinical practice, the decision to intubate is usually based on several clinical markers, including blood oxygenation [17], and may differ across institutions [18]. Furthermore, based on experimental [19, 20] and observational data [5, 6], a so-called “early approach” to invasive mechanical ventilation has been advocated for patients with non-COVID related ARDS [5]. Critically ill patients with COVID-19 often have profound hypoxemia which may partially explain the extremely high use of invasive ventilatory support in this patient population. This scenario, combined with the sharp rise in the incidence of COVID-19, has led to an unprecedented pressure on healthcare systems [14, 15, 21–23].

Previous reports on the use of HFNO in patients with COVID-19 have been mainly limited by small sample sizes and the reporting of unadjusted effect estimates [24]. Whether HFNO decreases the need for invasive mechanical ventilation in these patients remains unknown. In this study, we aimed to estimate the effect of HFNO on ventilator-free days (VFDs), ICU length of stay and in-hospital mortality, when compared to an early intubation strategy in adult patients with COVID-19 related acute respiratory failure. Our overall aim is to better inform the use of non-invasive oxygenation/ventilation strategies and the rational allocation of invasive mechanical ventilation.

## Methods

### Study design and setting

We conducted a prospective, multicentre, cohort study of consecutive patients with COVID-19 associated acute respiratory failure admitted to 36 hospitals from Spain and Andorra (see Supplementary file) [16]. The study was approved by the referral Ethics Committee of Hospital Clínic, Barcelona, Spain (#HCB/2020/0399), and conducted according to the amended Declaration of Helsinki. This report follows the “Strengthening the Reporting of Observational Studies in Epidemiology (STROBE)” guidelines for observational cohort studies [25]. Gathering of data is ongoing and as of August 13, 2020, a total of 1,129 patients have been included. A preliminary communication was presented as an abstract at the annual European Respiratory Society conference in September 2020 [26].

### Study population

We included adult patients (≥ 18 years old) admitted to the ICU between March 12 and August 13, 2020. Patients were included if they had positive confirmatory nasopharyngeal or pulmonary tract sample and received support with either HFNO or intubation on the first day of ICU admission. Main exclusion criteria were intubation outside the ICU, a PaO_2_/FiO_2_ ratio > 300 mmHg, a respiratory rate on day 1 > 35 breaths/min, a Glasgow Coma Score < 13, and pH < 7.25 [18, 27]. The rationale for the aforementioned eligibility criteria was based on a population that (with equipoise) could theoretically be randomized to a strategy of early intubation or HFNO in the first 24 h of critical illness, under the framework of a target randomized trial (Additional file 1: e-Table 1) [28]. The final analytical cohort was obtained by propensity score matching based on potential confounders measured at baseline.

**Table 1:** Baseline characteristics of the matched sample of adult patients with COVID-19 related acute respiratory failure

Covariate	Early intubation (N = 61)	HFNO (N = 61)	SMD
*Demographic characteristics*			
Age, *years—*mean (SD)	61 (11)	62 (11)	0.06
Female gender, n (%)	36 (48)	27 (40)	0.14
BMI, *kg/m2 –* mean (SD)	28.8 (4.3)	28.8 (5.5)	0.01
Time to ICU admission*, days –* median [IQR]	2 [1–4]	2 [1–4]	0.11
*Baseline comorbid disease*			
Number of comorbidities – median [IQR]	1 [0–1]	1 [0–2]	0.00
Immunosupression, (n, %)	2 (3.3)	4 (6.6)	0.15
Active cancer, (n, %)	0 (0)	6 (9.8)	0.47
*Initial severity of disease*			
SOFA score—median [IQR]	5 [3–7]	4 [4–7]	0.00
Glasgow coma score—median [IQR]	15 [15]	15 [15]	0.41
APACHE II score—median [IQR]	11 [9–14]	10 [9–113]	0.11
PaO_2_:FiO_2_ ratio—mean (SD)	117 (51)	121 (49)	0.09
Respiratory rate, r*pm—*mean (SD)	25 (5)	25 (5)	0.04
Oxygen saturation, *%—*mean (SD)	88 (7)	89 (6)	0.09
ROX index—median [IQR]	4.4 [3.4–6.4]	5 [4–6.2]	0.25
PaCO_2_, *mmHg*—mean (SD)	37 (8)	38 (12)	0.02
Gas flow, L/min—mean (SD)	–	55 (12)	–
FiO_2_, *%*—mean (SD)	79 (18)	72 (16)	0.45
Heart rate (bpm)—mean (SD)	81 (18)	82 (15)	0.03
Systolic blood pressure (mmHg)—mean (SD)	128 (21)	124 (18)	0.21
Use of steroids, n (%)	47 (77)	45 (73.8)	0.08
*Laboratory values*			
pH—mean (SD)	7.4 (0.1)	7.44 (0.06)	0.66
Creatinine, *mg/dL*—mean (SD)	1.0 (0.8)	1.0 (0.7)	0.01
Bilirrubin, *mg/dL*—mean (SD)	0.7 (0.5)	0.7 (0.3)	0.01
Lactate, *mmol/L*—mean (SD)	0.3 (0.6)	0.4 (0.7)	0.13
D-dimer, *U/L*—mean (SD)	4025 (11,944)	2235 (4724)	0.19
Leucocyte count, *10^9/L*—mean (SD)	8.1 (3.6)	8.3 (4.8)	0.04
Lymphocyte count, *10^9/L*—mean (SD)	0.7 (1.0)	0.7 (0.5)	0.09
Platelet count, *10^12/L*—mean (SD)	223 (88)	241 (126)	0.16

HFNO: high-flow nasal oxygen; IQR: interquartile range; SD: standard deviation; SMD: standardized mean difference; PaCO_2_: arterial pressure of carbon dioxide; FiO_2_: inspired oxygen fraction; ROX: ratio of oxygen saturation to FiO_2_, divided by respiratory rate: (Saturation/FiO_2_)/Respiratory rate. APACHE: Acute Physiology And Chronic Health Evaluation; SOFA: Sequential Organ Failure Assessment

### Data collection

Patients’ characteristics were collected prospectively according to a previously standardized common protocol. Each investigator had a personal username/password and entered data into a specifically pre-designed online data acquisition system (CoVid19.ubikare.io) endorsed and validated by the Spanish Society of Anesthesiology and Critical Care (SEDAR) [29]. Patient confidentiality was protected by assigning a de-identified code. Recorded data included patients’ demographics [age, gender, body mass index (BMI)], comorbidities, time from onset of symptoms and from hospital admission to initiation of respiratory support and vital signs (temperature, mean arterial pressure, heart rate), laboratory parameters (complete blood count, coagulation tests, electrolytes, creatinine) and severity assessment scales such as the Sequential Organ Failure Assessment (SOFA) [30] and Acute Physiology and Chronic Health Evaluation II (APACHE II) scores [31]. Site investigators collected what they considered to be the most representative data of each day from ICU admission to ICU discharge. Patients were followed up until hospital discharge to assess for in-hospital mortality.

### Study exposure and outcomes

The main exposure was the use of HFNO as the initial oxygenation strategy in the first 24 h (conservative group), and the comparison was the use of invasive mechanical ventilation in the first 24 h (early intubation group) [6]. Because data were collected once per day and the duration of HFNO use was not recorded, patients that were switched from HFNO to invasive-mechanical ventilation on day 1 were considered as part of the early intubation group [6]. We considered that, in these patients, the HFNO use may have been too short to have a meaningful effect in a patient’s outcome [6]. The decision to intubate was left at the discretion of the treating physicians at each participating centre. The primary outcome of interest was VFDs at day 28, calculated as 28 *minus* the days that a particular patient remained mechanically ventilated [32]. To account for the competing risk of death, deceased patients were considered to have 0 VFDs. Secondary outcomes included ICU length of stay, intubation rate and all-cause in-hospital mortality (and up to 60 days). A subgroup analysis considering patients intubated early (on day 1) versus those intubated late (from day 2 and onwards) was performed.

### Statistical analysis

Demographics, comorbidities, vital signs and laboratory markers at ICU admission were compared between both treatment groups using standardized mean differences. To account for potential confounding of the effects of HFNO on all outcomes of interest, we performed a propensity-score matched analysis [33]. Specifically, we built a multivariable logistic regression model to estimate the log-odds of receiving HFNO on the first day of ICU. The criteria to include variables in this model were based on those potentially affecting the likelihood of outcome occurrence and receipt of study treatments [34] and were performed based on subject matter knowledge with the help of a direct acyclic graph (DAG) (e-Fig. 1 in Additional file 1) [35, 36]. Selected variables included gender, APACHE II, SOFA, Glasgow Coma Scale, systolic blood pressure, pH, respiratory rate, arterial partial pressure of carbon dioxide (PaCO_2_), body mass index (BMI), creatinine, bilirubin, platelet, leucocyte and lymphocyte count, lactate, immunosuppression and hospital group (divided into quartiles based on the proportion of patients receiving intubation from the total). The matching procedure was conducted on a 1:1 fashion without replacement and with the calliper of the logit (propensity score) set at 0.2 [33]. Proper adjustment was assessed with standardized mean differences (SMD) in the matched population, and covariate imbalance defined using a SMD > 0.2 threshold [33]. Missing data on important confounders were handled using multiple imputations with a Monte Carlo Markov chain method (details in Additional file 1) [37].

**Fig. 1:**
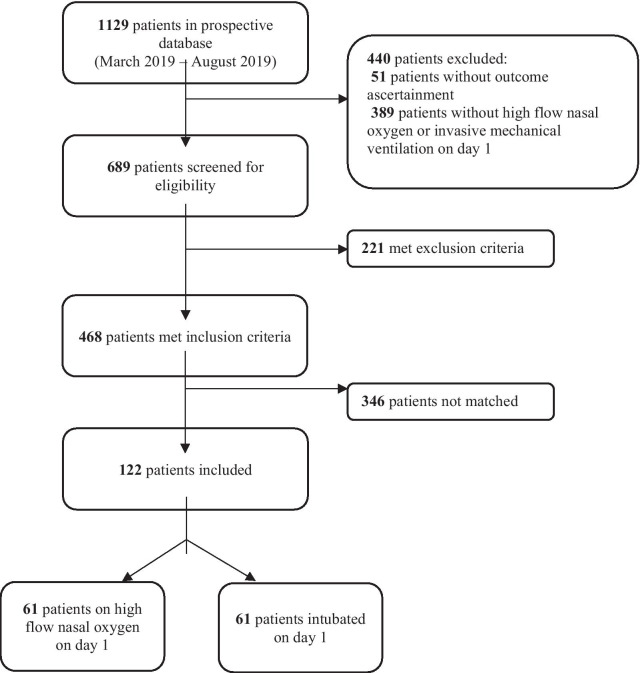
Study flowchart

Once the matched cohort was constructed and after balance assessment, we used simple linear regression to assess mean differences in VFDs at 28 days and ICU length of stay (in days) between treatment groups. For all-cause in-hospital mortality, we used generalized linear models (with identity link and binomial distribution) to estimate risk differences and a crude logistic regression model to estimate odds ratios. For all models, 95% confidence intervals (CI) were constructed based on robust standard errors to account for the matching procedure.

### Sensitivity analyses

Several sensitivity analyses were performed to assess the robustness of our findings for the study outcomes. First, we performed a complete case analysis, excluding patients that had any missing data on the selected variables to construct the propensity score. Second, we repeated our primary analysis for the treatment effect by adjusting for those baseline variables that were not balanced (i.e., SMD > 0.2) by our matching procedure [38]. Third, given that treatment assignment was not random, both residual and unmeasured confounding remain possible. Hence, we estimated the E-value as a way to determine the association between an unmeasured confounder with both the exposure (HFNO) and outcome that would fully explain the estimated effect (see details in Additional file 1). Fourth, we changed our exposure classification, keeping patients who initially received HFNO and switched within the 24-h window to invasive mechanical ventilation as part of the conservative strategy (HFNO). This was done to evaluate whether the initial classification yielded optimistic estimates by assigning sicker patients with early HFNO failure to the early intubation group. Finally, we assessed the modification of the effect of HFNO on the primary outcome of interest according to baseline severity measured by the PaO_2_/FiO_2_ ratio. For subgroup analysis, we used Wilcoxon rank-sum test and Fisher’s test as appropriate.

We used a threshold of 0.05 for statistical significance. All reported tests are two-sided. The R software (R Foundation for Statistical Computing, Vienna, Austria; packages mice, lme4 and sjstats packages) and STATA v.14.2 were used for all analysis. The E-value was computed using a freely available online calculator (www.evalue-calculator.com). Graphs were constructed using BioRender.com.

## Results

### Study population

From March 12 to August 13, 2020, 468 critically ill patients with COVID-19 patients fulfilled the inclusion criteria for the present study (Fig. 1). Three-hundred and twelve (67%) patients were intubated on day 1 (37 of them after a HFNO trial). The remaining 156 patients received HFNO, of whom 49 (31%) received intubation from day 2 and onward. Baseline characteristics for the entire population (before matching) are shown in Additional file 1: e-table 2. After propensity score matching, 61 patients in each group were included. Overall, we observed adequate balance between most of baseline characteristics with the exception of baseline ROX index, systolic blood pressure, Glasgow Coma Scale, pH, inspired oxygen fraction (FiO_2_) and active cancer (Table 1).

### Study outcomes

**Fig. 2:**
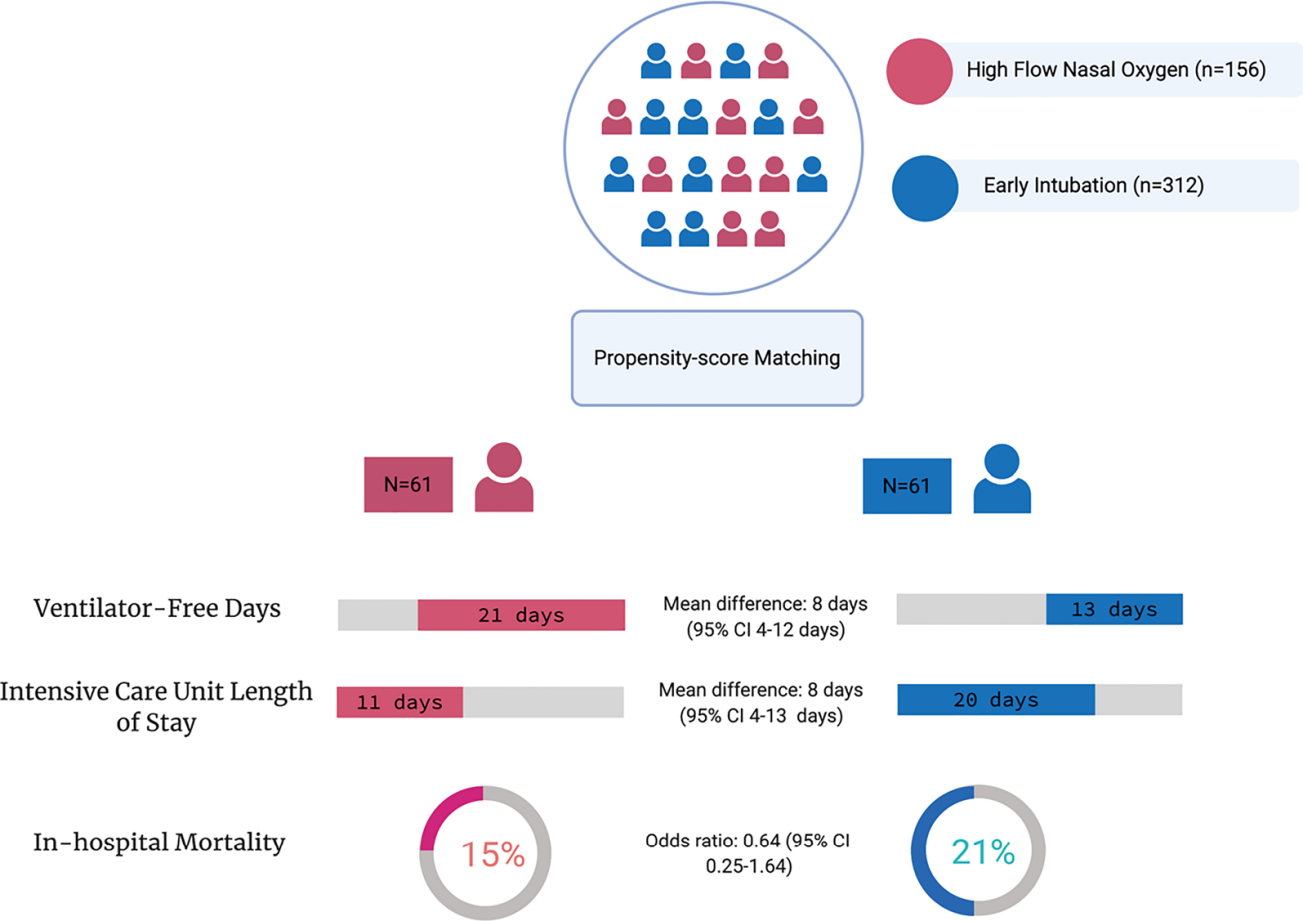
Effect of a conservative approach (use of high-flow nasal oxygen) compared to early intubation on main outcomes of interest for patients with COVID-19 associated acute respiratory failure. Difference is expressed as mean difference for continuous variables or absolute risk difference for in-hospital mortality. In-hospital mortality in both groups expressed as cumulative incidence. CI: Confidence interval. HFNO: high-flow nasal oxygen. (1) Cumulative incidence and cumulative incidence difference (i.e., risk difference; 95% CI) reported for both groups. Results for ventilator-free days and intensive care unit length of stay were rounded up or down to the closest whole number

When compared to an early intubation strategy, the use of HFNO was associated with an increase in VFDs (mean difference 8.0 days; 95% CI 4.4 to 11.7 days), and a reduction in ICU length of stay (mean difference -8.2 days; 95% CI -12.7 to -3.6 days). Intubation rate was 38% in the conservative group (compared to an expected 100% in the early intubation group). No difference was observed in all-cause in-hospital mortality between groups (OR 0.64; 95% CI 0.25 to 1.64) (Fig. 2).

### Sensitivity analysis

All sensitivity analysis yielded similar results to the main estimates (Additional file 1: e-table 3). Specifically, in the complete-case analysis, the use of HFNO remained associated with an increase in VFDs (mean difference 6.8 days; 95% CI 1.5 to 12.1 days) and shorter ICU length of stay (mean difference 12.3 days; 95% CI 19.8 to 4.7). No difference was observed in all-cause hospital mortality (OR 1.64; 95% CI 0.40 to 6.66). Furthermore, after adjusting for imbalanced covariates, namely the presence of an active cancer, Glasgow Coma Scale, ROX index and FiO_2_, the use of HFNO remained associated with an increase in VFDs (mean difference 7.7 days; 95% CI 3.6 to 11.9) and shorter ICU length of stay (mean difference -9.4; 95% CI -14.7 to -4.0) when compared to an early intubation approach. No difference was observed in all-cause hospital mortality (OR 0.75; 95% CI 0.22 to 2.55). The estimated E-value for the primary analysis for the effects of HFNO on VFDs was 3.28 (e-Fig. 2 in Additional file 1). Finally, no modification of the effects of HFNO on VFDs was evident by baseline PaO_2_/FiO_2_ ratio (Additional file 1: e-table 4).

### Subgroup analysis by the time of intubation

Of the 61 included patients who initially received a conservative strategy with HFNO, 23 (38%) were intubated from day 2 onward. When compared to patients intubated early in their ICU course, VFDs (median 10 vs 15 days, p = 0.88), ICU length of stay (12 vs 17 days, p = 0.41) and in-hospital mortality (26% vs 21%, p = 0.77) did not differ (Additional file 1: Table S5).

## Discussion

In this multicentre observational cohort study of 122 matched, critically ill adult patients with COVID-19 associated acute hypoxemic respiratory failure, the use of HFNO was associated with an increase in VFDs and shorter ICU length of stay when compared to an early intubation strategy. No significant differences were evident in all cause in-hospital mortality.

The COVID-19 pandemic has unveiled the ongoing uncertainty and resulting discussions as to whether patients presenting with significant hypoxemia should undergo an early intubation strategy or whether, on the contrary, a conservative non-invasive approach could be offered [39–41]. Importantly, the benefits of the use of non-invasive oxygenation/ventilation strategies in the context of acute respiratory failure need to be balanced against the risk of treatment failure, given its potential association with worse clinical outcomes in non-COVID-19 populations [6, 7]. The results of this analysis are consistent with other studies showing potential beneficial effects of HFNO in the context of COVID-19 associated acute respiratory failure [42] and reinforce recent evidence showing that HFNO was associated with a reduced risk of intubation in this patient population [43].

To the best of our knowledge, this is the first study specifically comparing HFNO with an early intubation strategy. This study provides additional evidence that in a population with similar baseline characteristics and a potential to be randomized to any of these interventions, the use of HFNO may be associated with an increase in VFDs and shorter duration of ICU length of stay without any significant difference in mortality. Of note, on average, patients receiving early intubation in our cohort were sicker at baseline, as assessed by higher SOFA and APACHE II scores. However, matching achieved good balance in most of the covariates assessed and our results were robust to a variety of sensitivity analysis, including a secondary analysis in which adjustment by imbalanced variables was performed. Further, in the matched population, hypoxemia was profound despite the use of high FiO_2_ and the benefit spanned across the entire spectrum of PaO_2_/FiO_2_ values, as shown by our sensitivity analysis stratifying by oxygenation levels. This finding suggests that moderate-to-severely hypoxemic patients affected by COVID-19 may benefit from HFNO and that HFNO could potentially decrease the need and duration of mechanical ventilation and ICU length of stay without a negative impact in hospital mortality.

Several limitations need to be taken into account when interpreting the findings of our study. First, since treatment was not randomly allocated, both residual and unmeasured confounding are likely even after careful covariate adjustment. Nonetheless, the moderately robust E-value, together with a pre-planned emulation of a target trial increase the confidence in our study findings. Second, the use of VFDs as the primary outcome could be considered to favour upfront the HFNO group, given that a significant proportion of patients on HFNO was not subsequently intubated. As reported elsewhere, this endpoint encompasses both the time spent on mechanical ventilation as well as mortality and, for any given value in a population, both components should be provided to avoid misleading conclusions [32]. Given the similar mortality risk in both groups, the observed differences in VFDs between groups may be mostly driven by a reduction in the need for intubation among those initially treated with HFNO or, as previously stated, may also be due to unmeasured or residual confounding (e.g., patients who are sicker at baseline predominantly receive early invasive mechanical ventilation and have lower VFDs than those who have less severe disease and initially receive HFNO). Although an untestable assumption, our E-value and robustness to a variety of sensitivity analysis may point towards a potential causal effect rather than confounding as the main explanation for this finding. Explicitly, if both the HFNO and early invasive mechanical ventilation groups are considered comparable at baseline (e.g., regarding their initial severity), then the reduction in VFDs remains informative as it points towards a reduction in intubation as the likely mechanistic pathway—something that has been shown elsewhere in the broad population of critically ill patients with acute respiratory failure. Third, sample size was limited due to the inability to match a significant proportion of patients. This has to be understood in the context of populations differing significantly (the overall group of patients receiving early intubation was sicker) and the choice of very strict adjustment criteria to improve the precision of the estimates. In light of this fact, clinicians should keep in mind that the potential benefit of the treatment might be limited to patients with similar characteristics to the matched cohort (moderate-to-severe hypoxemic patients without concomitant non-pulmonary organ dysfunction) although two additional sensitivity analysis using the whole population also favoured HFNO. Fourth, missing information was present for several covariates of interest possibly resulting in both information bias and residual confounding. However, our multiple imputation-based results were consistent with the complete case analysis. Fifth, misclassification of relevant covariates and potential predictors is also likely. However, a concise operational manual was provided to all researchers at the study initiation, and two investigators checked for the accuracy of the data and unreliable values for all included patients. Sixth, criteria for intubation were not uniformly defined, and hence, the reported rate of failure and the effect of HFNO may not be generalizable to other settings with distinct clinical practice patterns. Sixth, code status at admission was not recorded and this might have impacted the rate of intubation in the conservative group. Indeed, despite achieving good balance between groups after matching, the presence of cancer was still more common in the conservative group. However, the mortality risk was similar across groups, our results were robust across sensitivity analysis adjusting for imbalanced covariates, and the intubation risk in the conservative group was 38%, which is in line with previous reports [27]. Finally, this study cannot offer any information regarding a potential increased risk of COVID-19 infection in healthcare professionals with HFNO use although studies have not been able to show an increase risk with this therapy [44].

## Conclusion

In this observational study of 122 matched adult critically-ill patients with COVID-19-associated acute respiratory failure receiving either HFNO or early intubation upon ICU admission, the use of HFNO was associated with increased VFDs and a reduction in the duration of ICU stay, with no differences in all-cause in-hospital mortality. However, caution is warranted before drawing definite conclusions since, in the whole population, those intubated early were sicker, and even the most thorough statistical adjustment cannot eliminate confounding entirely. Future studies should corroborate our findings, as a way of optimizing the ventilation strategy for patients with COVID-19 associated acute respiratory failure.

## Supplementary information

**Additional file 1.** It contains further information on methodology as well as 4 tables and 2 figures.

## Data Availability

By request to the corresponding author.

## Abbreviations

APACHE: Acute physiological and Chronic Health disease Classification System
ARDS: Acute respiratory distress syndrome
ARF: Acute respiratory failure
BMI: Body mass index
CI: Confidence interval
COVID-19: Coronavirus Disease 19
CRP: C-Reactive protein
DAG: Direct acyclic graph
HFNO: High-flow nasal oxygen therapy
ICU: Intensive care unit
IQR: Interquartile range
LOS: Length of stay
MV: Mechanical ventilation
PaO_2_/FiO_2_: Partial pressure of arterial oxygen to inspiratory oxygen fraction ratio
P-SILI: Patient self-inflicted lung injury
OR: Odds ratio
RR: Respiratory rate
SBP: Systolic blood pressure
SD: Standard deviation
SMD: Standardized mean difference
SOFA: Sequential organ failure assessment
SpO2: Peripheral oxyhaemoglobin saturation
STROBE: Strengthening the Reporting of Observational Studies in Epidemiology
VFD: Ventilator-free day
