# Supplementary material for: High-flow nasal oxygen in patients with COVID-19-associated acute respiratory failure
Source: Crit Care. 2021 Feb 11;25:58. doi: 10.1186/s13054-021-03469-w (PMC7876530; doi:10.1186/s13054-021-03469-w)
Supplement: Supplementary file 1 — Additional file 1. It contains further information on methodology as well as 4 tables and 2 figures. [file 13054_2021_3469_MOESM1_ESM.docx]

**High flow nasal oxygen in patients with COVID-19 associated acute respiratory failure**

Ricard Mellado-Artigas (MD)^1^, Bruno L. Ferreyro (MD)^2^, Federico Angriman (MD, MPH)^3,4^, María Hernández-Sanz (MD)^5^, , Egoitz Arruti (PhD)^6^, Antoni Torres (MD, PhD)^7,8,9^ , Jesús Villar (MD, PhD)^8,10^, Laurent Brochard (MD, PhD)^3, 11^, Carlos Ferrando (MD, PhD)^1,8^, for the COVID-19 Spanish ICU Network.

From

1. *Department of Anesthesiology and Critical Care, Hospital Clínic, Institut D'investigació August Pi i Sunyer, Barcelona, Spain;*
2. Department of Medicine, Sinai Health System and University Health Network, Toronto, Canada.
3. *Interdepartmental Division of Critical Care Medicine, University of Toronto, Canada;*
4. *Department of Critical Care Medicine. Sunnybrook Health Sciences Centre. Toronto, Canada;*
5. *Department of Anesthesiology and Critical Care, Hospital de Cruces, Vizcaya, Spain;*
6. *Ubikare Technology, Vizcaya, Spain;*
7. *Department of Respirology, Hospital Clínic, Institut D'investigació August Pi i Sunyer, Barcelona, Spain;*
8. *CIBER de Enfermedades Respiratorias, Instituto de Salud Carlos III, Madrid, Spain;*
9. *CIBERESUCICOVID, Instituto de Salud Carlos III, Madrid, Spain;*
10. *Multidisciplinary Organ Dysfunction Evaluation Research Network, Research Unit, Hospital Universitario Dr. Negrin, Las Palmas de Gran Canaria, Spain.*
11. *Keenan Research Centre for Biomedical Science, St Michael’s Hospital, Toronto, Canada*

#### SUPPLEMENTARY APENDIX

[1. Further details on statistical analysis 2](#_Toc51221032)

[1.a. Selection of variables for adjustment of confounding 2](#_Toc51221033)

[1.b. Multiple data imputation 2](#_Toc51221034)

[1.c. Estimation of E-value 3](#_Toc51221035)

[2. Figures and tables 4](#_Toc51221036)

[E-figure 1. Directed acyclic graph (DAG) 5](#_Toc51221037)

[E-FIGURE 2. E-value calculation for primary outcome of interest (ventilator free days) 6](#_Toc51221038)

[E-TABLE 1. Overall cohort design and differences with a pre-planned pragmatic target randomized trial of high flow nasal oxygen compared to an early intubation strategy for patients with COVID-19 associated acute respiratory failure. 7](#_Toc51221039)

[E-TABLE 2. Characteristics of the baseline study sample before matching. 8](#_Toc51221040)

[E-TABLE 3. Summary of sensitivity analysis 9](#_Toc51221041)

[E-TABLE 4. Subgroup analysis by baseline PaO_2_:FiO_2_ ratio 10](#_Toc51221042)

[3. REFERENCES 11](#_Toc51221043)

**4. SPANISH COVID-19 ICU NETWORK……………………………………………………………………………………………12**

# 1.- Further details on statistical analysis

## 1.a. Selection of variables for adjustment of confounding

Covariate adjustment poses the risk to induce selection bias if conditioning is performed on variables that act as colliders. The structural relationship between the exposure of interest and outcome, including all other potential covariates can be depicted using a direct acyclic graph (DAG)^1–4^. This form of causal diagram is constructed from subject matter knowledge including previous research and describes the roadmap of causal connections between variables of interest. Through the assessment of relationships between variables, DAGs help to identify potential sources of bias and how to control for them when estimating causal associations^4^. The DAG used for this study is depicted on supplementary figure 1. With the DAG, we identified six nodes to be controlled during matching; namely the degree of respiratory failure and extrapulmonary involvement, the number of comorbidities, the age, and the ICU were a particular patient was admitted. For 5 out of 6 nodes we were able to identify variables that could be used to control for confounding such as age, the number of comorbidities and type of comorbidities, oxygenation and severity scores or vital signs as well as laboratory markers. Gender was included since some reports suggest higher severity in male individuals. Covariate adjustment was assessed using standardized mean differences.

## 1.b. Multiple data imputation

Overall, 881 (4%) variable-observation pairs were missing. For that reason, multiple imputation using a Monte Carlo Markov chain method was carried out with the mice package in R^5,6^. For every missing value, we created 1000 imputations and final imputed values for each missing observation were later calculated as the median of all the imputations. There was no missing data in the study exposure.

## 1.c. Estimation of E-value

Unmeasured confounding represents a major concern when estimating causal effects in observational studies thus a metric that calculates how strong an unmeasured confounder should be to overturn the reported results is advisable. The E-value, which is presented in the risk ratio scale (with values from 1 to infinite), represents the strength of association that a confounder should present with both the exposure and the outcome to explain away the estimated findings^7^. We performed the calculation of the e-value for the primary outcome using the e-value calculator: <https://www.evalue-calculator.com>.

# 2.- Figures and tables

## Figure S1. Directed acyclic graph (DAG)


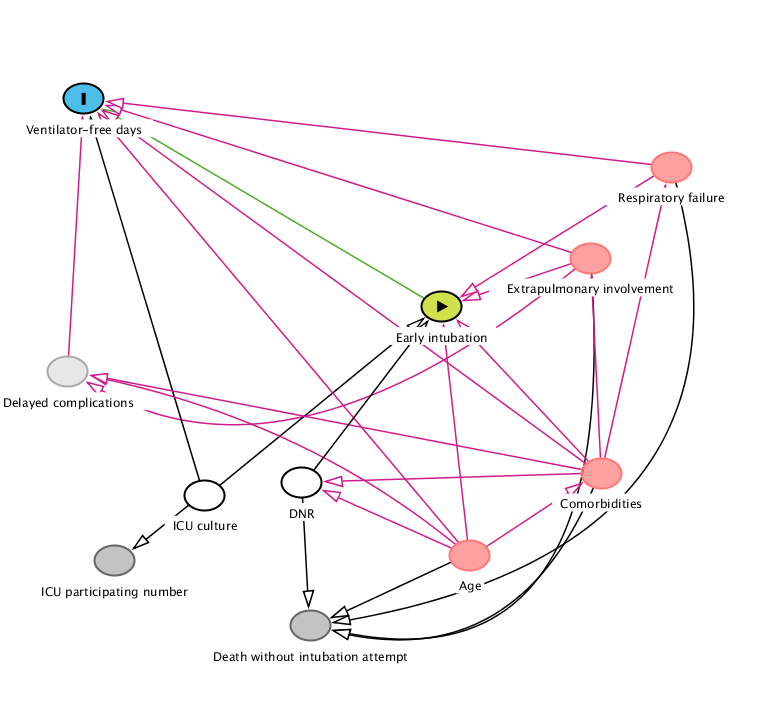


Direct acyclic graph (DAG) depicting variables of interest (dots) and causal relationships (arrows). Top left in blue: primary outcome. Green dot in the middle: exposure. The relationship of interest in this study is the one connecting both points. In red: predictor variables that affect both the exposure and the outcome in which direct data was available. In white: unobserved predictor variables. In dark grey: surrogate markers for unobserved variables. In light grey: the presence of delayed complications (after day 1) could influence the outcome but not the treatment received (either early intubation or not), hence it was not adjusted for.

## Figure S2. E-value calculation for primary outcome of interest (ventilator free days)


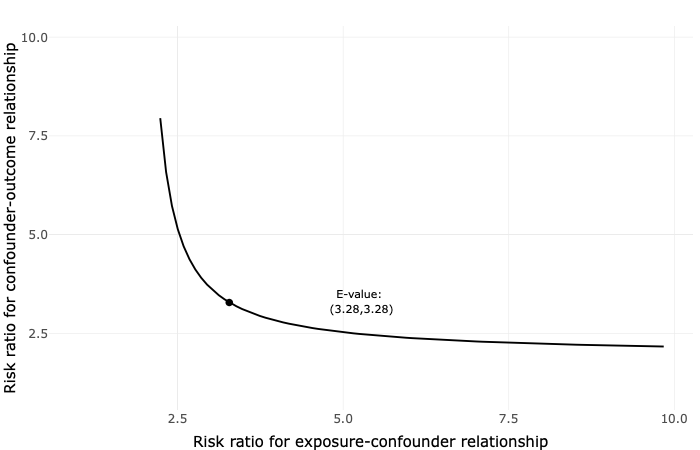


The E-value shows, on the risk ratio scale, the strength of association between the unmeasured confounder and both the exposure and the outcome that would explain away the reported association.

Specifically, for the estimated effect of high flow nasal oxygen on ventilator free days, a risk ratio of 3.28 for both the confounder-outcome relationship and the exposure-confounder relationship would be needed to draw the results to the null value. This shows moderate robustness to potential unmeasured confounding.

## Table S1. Overall cohort design and differences with a pre-planned pragmatic target randomized trial of high flow nasal oxygen compared to an early intubation strategy for patients with COVID-19 associated acute respiratory failure.

| **Component** | **Target trial** | **Emulation** |
| --- | --- | --- |
| Eligibility | Adult patients (18 years or older) with COVID-19 related acute respiratory failure suitable for either a conservative or early intubation strategy; PaO_2_/FiO_2_ ratio < 300 mmHg; a respiratory rate on day 1 < 35 breaths/min; Glasgow Coma Score <13 and pH ≤7.25 | Adult patients (18 years or older) with COVID-19 related acute respiratory failure suitable for either a conservative or early intubation strategy; PaO_2_/FiO_2_ ratio < 300 mmHg; a respiratory rate on day 1 < 35 breaths/min; Glasgow Coma Score <13 and pH ≤7.25 |
| Treatment strategies | High flow nasal oxygen or invasive mechanical ventilation. Treatment started after randomization. | High flow nasal oxygen or invasive mechanical ventilation. Treatment within the first day of intensive care unit admission. |
| Treatment assignment | Permuted randomization scheme with varying block sizes from 4 to 8 | We will assume that patients were “randomly assigned” within levels of included confounders (i.e., conditional exchangeability). |
| Follow-up | In-hospital mortality; up to 60 days | In-hospital mortality; up to 60 days |
| Primary end point | Ventilator free days | Same as target trial |
| Secondary end point | Intensive care unit length of stay  All-cause in hospital mortality | Intensive care unit length of stay  All-cause in hospital mortality |
| Causal contrast of interest | Intention to treat effect | Observational analogue of the intention to treat effect |
| Statistical analysis | Survival curves will be estimated according to the Kaplan–Meier procedure. Crude generalized linear models for primary and secondary outcomes. | Same as target trial |

## Table S2. Characteristics of the baseline study sample before matching.

| **Baseline characteristic** | **Early intubation**  **(N=312)** | **High flow nasal canula**  **(N=156)** | **p value** |
| --- | --- | --- | --- |
| Time to ICU admission*, days –* median [IQR] | 2 [1-4] | 2 [1-4] | 0.77 |
| Age, mean (SD) | 62.3 (10.9) | 60.6 (12.7) | 0.10 |
| Female gender | 101 (32.4%) | 43 (27.6%) | 0.29 |
| Body mass index, mean (SD) | 29.4 (4.9) | 28.1 (4.3) | < 0.01 |
| N. of comorbidities, median (IQR) | 1 (0-2) | 1 (0-2) | 0.93 |
| Immunosuppression | 13 (4.2%) | 10 (6.4%) | 0.29 |
| Cancer | 10 (3.2%) | 8 (5.1%) | 0.31 |
| SOFA, median (IQR) | 7 (5-8) | 4 (3-5) | <0.01 |
| Glasgow, median (IQR) | 15 (15-15) | 15 (15-15) | 0.01 |
| APACHE, median (IQR) | 12 (10-14) | 10 (7-13) | <0.01 |
| PaO2:FiO2 ratio, mean (SD) | 121.2 (58.0) | 122.7 (48.5) | 0.78 |
| Respiratory rate, mean (SD) | 24.7 (6.0) | 25.3 (5.2) | 0.26 |
| Sat, mean (SD) | 87.2 (9.2) | 90.4 (4.9) | < 0.01 |
| ROX index, median (IQR) | 4 (3.2-6) | 5.1 (4.1-6.6) | < 0.01 |
| PaCO2, mean (SD) | 44.7 (12.9) | 35.4 (9.4) | < 0.01 |
| Gas flow during HFNO, mean (SD) |  | 54 (12) | - |
| FiO2, mean (SD) | 84.6 (18.2) | 71.2 (16.8) | < 0.01 |
| Heart rate, mean (SD) | 84.8 (19.2) | 81.2 (15.4) | 0.04 |
| Systolic BP, mean (SD) | 120.9 (21.5) | 126.8 (18.3) | < 0.01 |
| PH, mean (SD) | 7.4 (.1) | 7.5 (.1) | < 0.01 |
| Creatinine, mean (SD) | 1.0 (.6) | 1.1 (1.0) | 0.19 |
| Bilirubin, mean (SD) | 0.9 (1.5) | 0.9 (1.9) | 0.92 |
| Lactate, mean (SD) | 0.3 (1.0) | 0.4 (.6) | 0.19 |
| D-Dimer, mean (SD) | 4486 (11467) | 2029 (3616) | < 0.01 |
| Leucocytes, mean (SD) | 10.3 (5.9) | 8.4 (5.3) | < 0.01 |
| Lymphocytes, mean (SD) | 0.8 (.8) | 0.7 (0.6) | 0.23 |
| Platelets, mean (SD) | 247 (103) | 250 (112) | 0.78 |
| **Outcomes** |  |  |  |
| Ventilation-free days, median (IQR) | 13 (0-21) | 28 (22-28) | <0.01 |
| ICU length of stay, median (IQR) | 16 (8-25) | 7 (4-12) | <0.01 |
| In-hospital mortality, n (%) | 99 (32%) | 20 (13%) | <0.01 |

Categorical variables presented as count (%). PaCO2: arterial pressure of carbon dioxide. FiO_2_: inspired oxygen fraction. ROX: ratio of oxygen saturation to FiO_2_, divided by respiratory rate: (Saturation/FiO2)/Respiratory rate. Continuous variables are expressed as medians (interquartile range); APACHE: Acute Physiology And Chronic Health Evaluation; SOFA: Sequential Organ Failure Assessment.

## Table S3. Summary of sensitivity analysis

|  | **Mean difference (95% CI)** | | | |
| --- | --- | --- | --- | --- |
| **Outcome of interest** | Adjusting for imbalance in matched sample | Adjusting on propensity score (entire sample) | Complete case analysis (matched analysis) | Adjusting on propensity score and considering HFNO at baseline (disregarding day 1) |
| Ventilator free days | 7.7 (3.6, 11.9) | 7.3 (4.5, 10.1) | 6.8 (1.4, 12.1) | 5.0 (2.7, 7.4) |
| Intensive care unit length of stay - days | -9.4 (-14.7, -4.0) | -8.0 (-11.3, -4.7) | -12.3 (-19.8, -4.7) | -6.0 (-9.2, -2.8) |
| In-hospital mortality^1^ | 0.75 (0.22, 2.55) | 0.69 (0.33 – 1.44) | 1.64 (0.40, 6.66) | 0.77 (0.43, 1.36) |

SMD: standardized mean difference VFD: Ventilator-free days. Continuous variables are expressed as median difference while in-hospital mortality is expressed as odds ratio. 95% CI: 95% confidence interval.

1. **Results for in hospital mortality shown as odds ratio (95%CI)**

## Table S4. Subgroup analysis by baseline PaO_2_:FiO_2_ ratio

| **Effect of HFNO vs. early intubation** | **Ventilator free days**  **[Mean difference (95% CI)]^1^** |
| --- | --- |
| PaO_2_:FiO_2_ ratio < 200 | 6.3 (-10.1 to 22.8) |
| PaO_2_:FiO_2_ ratio >= 200 | 8.2 (-1.2 to 17.6) |

1. P for interaction = 0.97.

HFNO: high flow nasal oxygen.

#

## Table S5. Characteristics of patients intubated early and late in the matched population

| **Baseline characteristic** | **Early intubation**  **(N=61)** | **Late intubation**  **(N=23)** | **p value** |
| --- | --- | --- | --- |
| Time to ICU admission*, days –* median [IQR] | 2 [1-4] | 2 [0-4] | 0.77 |
| Age, mean (SD) | 61 (11) | 63 (9) | 0.46 |
| Female gender, n (%) | 36 (48) | 9 (39%) | 0.81 |
| Body mass index, mean (SD) | 28.8 (4.3) | 29.5 (4.9) | 0.53 |
| N. of comorbidities, median (IQR) | 1 [0-1] | 1 (0-2) | 0.74 |
| Immunosuppression | 2 (3.3) | 1 (4.3%) | >0.99 |
| Cancer | 0 (0) | 0 (0%) | >0.99 |
| SOFA, median (IQR) | 5 [3-7] | 6 [4-8] | 0.39 |
| Glasgow, median (IQR) | 15 [15-15] | 15 [15-15] | 0.42 |
| APACHE, median (IQR) | 11 [9-14] | 10 [9-13] | 0.74 |
| PaO2:FiO2 ratio, mean (SD) | 117 (51) | 115 (51) | 0.83 |
| Respiratory rate, mean (SD) | 25 (5) | 27 (5) | 0.27 |
| Sat, mean (SD) | 88 (7) | 87 (8) | 0.49 |
| ROX index, median (IQR) | 4.4 [3.4-6.4] | 4.5 [3.3-5.9] | 0.91 |
| PaCO2, mean (SD) | 37 (8) | 37 (9) | 0.53 |
| FiO2, mean (SD) | 79 (18) | 75 (18) | 0.36 |
| Heart rate, mean (SD) | 81 (18) | 81 (14) | 0.94 |
| Systolic BP, mean (SD) | 128 (21) | 121 (19) | 0.14 |
| PH, mean (SD) | 7.4 (0.1) | 7.4 (0.1) | 0.23 |
| Creatinine, mean (SD) | 1.0 (0.8) | 1.1 (0.7) | 0.69 |
| Bilirubin, mean (SD) | 0.7 (0.5) | 0.6 (0.3) | 0.32 |
| Lactate, mean (SD) | 0.3 (0.6) | 0.4 (0.7) | 0.47 |
| D-Dimer, mean (SD) | 4025 (11944) | 1771 (2250) | 0.38 |
| Leucocytes, mean (SD) | 8.1 (3.6) | 8.3 (5.8) | 0.82 |
| Lymphocytes, mean (SD) | 0.7 (1.0) | 0.6 (0.4) | 0.52 |
| Platelets, mean (SD) | 223 (88) | 216 (156) | 0.79 |
| Use of steroids, n (%) | 47 (77) | 18 (78) | >0.99 |
| **Outcomes** |  |  |  |
| Ventilation-free days, median (IQR) | 15 (2-22) | 10 (0-23) | 0.88 |
| ICU length of stay, median (IQR) | 17 (12-24) | 12 (9-24) | 0.41 |
| In-hospital mortality, n (%) | 13 (21%) | 6 (26%) | 0.77 |

# 3.- REFERENCES

1. VanderWeele TJ, Hernán MA, Robins JM. Causal directed acyclic graphs and the direction of unmeasured confounding bias. *Epidemiology*. 2008;19(5):720-728. doi:10.1097/EDE.0b013e3181810e29

2. Etminan M, Collins GS, Mansournia MA. Using Causal Diagrams to Improve the Design and Interpretation of Medical Research. *Chest*. 2020;158(1):S21-S28. doi:10.1016/j.chest.2020.03.011

3. Hernán MA, Hernández-Díaz S, Robins JM. A Structural Approach to Selection Bias. *Epidemiology*. 2004;15(5):615-625. doi:10.1097/01.ede.0000135174.63482.43

4. Lederer DJ, Bell SC, Branson RD, et al. Control of confounding and reporting of results in causal inference studies. *Ann Am Thorac Soc*. 2019;16(1):22-28. doi:10.1513/AnnalsATS.201808-564PS

5. Sterne JAC, White IR, Carlin JB, et al. Multiple imputation for missing data in epidemiological and clinical research: potential and pitfalls. *BMJ*. 2009;338:b2393. doi:10.1136/bmj.b2393

6. van Buuren S, Groothuis-Oudshoorn K. mice: Multivariate imputation by chained equations in R. *J Stat Softw*. 2011;45(3):1-67. doi:10.18637/jss.v045.i03

7. Haneuse S, Vanderweele TJ, Arterburn D. Using the E-Value to Assess the Potential Effect of Unmeasured Confounding in Observational Studies. *JAMA - J Am Med Assoc*. 2019;321(6):602-603. doi:10.1001/jama.2018.21554

**4. Spanish ICU Network**

**Hospital Clínic de Barcelona**, Department of Anesthesiology and Critical Care: Marina Vendrell, Gerard Sánchez-Etayo, Amalia Alcón, Isabel Belda, Mercé Agustí, Albert Carramiñana, Isabel Gracia, Miriam Panzeri, Irene León, Jaume Balust, Ricard Navarro, María José Arguís, María José Carretero, Cristina Ibáñez, Juan Perdomo, Antonio López, Manuel López-Baamonde, Tomás Cuñat, Marta Ubré, Antonio Ojeda, Andrea Calvo, Eva Rivas, Paola Hurtado, Roger Pujol, Nuria Martín, Javier Tercero, Pepe Sanahuja, Marta Magaldi, Miquel Coca, Elena del Rio, Julia Martínez-Ocon, Paula Masgoret, Monserrat Tio, Angel Caballero, Raquel Risco, Lidia Gómez, Nicolás de Riva, Ana Ruiz, Beatriz, Tena, Sebastián Jaramillo, José María Balibrea, Francisco Borja de Lacy, Ana Otero, Ainitze Ibarzabal, Raquel Bravo, Anna Carreras, Daniel Martín-Barreda, Alfonso Jesús Alias, Mariano Balaguer, Jorge Aliaga, Alex Almuedo, Joan Ramón Alonso, Rut Andrea, Gerard Sergi Angelès, Marilyn Arias, Fátima Aziz, Joan Ramon Badía, Enric Barbeta, Toni Torres, Guillem Batiste, Pau Benet, Xavi Borrat, María Borrell, Ernest Bragulat, Inmaculada Carmona, Manuel Castellà, Pedro Castro, Joan Ceravalls, Oscar Comino, Claudia Cucciniello, Clàudia De Deray, Oriol De Diego, Paula De la Matta, Marta Farrero, Javier Fernández, Sara Fernández, Anna Fernández, Miquel Ferrer, Ana Fervienza, María Tallo Forga, Daniel Forné, Clàudia Galán, Andrea Gómez, Eduard Guasch, María Hernández- Tejero, Adriana Jacas, Beltrán Jiménez, Pere Leyes, Teresa López, José Antonio Martínez, Graciela Martínez-Pallí, Jordi Mercadal, Guido Muñoz, José Muñoz, Ricard Navarro, Josep María Nicolás, José Tomás Ortiz, Anna Peiró, Manuel Pérez, Esteban Poch, Margarida Pujol, Eduard Quintana, Bartomeu Ramis, Enric Reverter, Irene Rovira, Pablo Ruiz, Elena Sandoval, Stefan Schneider, Oriol Sibila, Carla Solé, Alex Soriano, Dolors Soy, M. Suárez, Adrián Téllez, Néstor David Toapanta, Antoni Torres, Xavier Urra. **Hospital Universitario Río Hortega**: César Aldecoa, Alicia Bordell, Silvia Martín, Judith Andrés. **Hospital Universitario Cruces:** Alberto Martínez Ruiz, Gonzalo Tamayo Medel, Iñaki Bilbao Villasante, Fernando Iturri Clavero, Covadonga Peralta Álvarez, Julia T. Herrera Díez, Andrea García Trancho, Iñaki Sainz Mandiola, Carmen Ruano Suarez, Angela Ruiz Bocos, Eneritz Urrutia Izagirre, Pablo Ortiz de Urbina Fernández, Naiara Apodaka López, Leire Prieto Molano, Eunate Ganuza Martínez, Iratxe Vallinas Hidalgo, Karmele de Orte Sancho, Celia González Paniagua, Gemma Ortiz Labrador, Mireia Pérez Larrañaga, Marta López Miguelez, Estíbaliz Bárcena Andrés, Erik Urutxurtu Laureano, Maria Jesús Maroño Boedo, Blanca Escontrela Rodríguez, Aitziber Ereñozaga Camiruaga, Deiene Lasuen Aguirre, Ainhoa Zabal Maeztu, Ane Guereca Gala, Iker Castelo Korro, Andrés Álvarez Campo, Alejandro Carcelen Viana, Alejandro Alberdi Enríquez, Xabier Ormazábal Rementeria, Alberto Sánchez Campos, Rosa Gutiérrez Rico, Pablo Barbier Damborenea, Marta Guerenabarrena Momeñe, Borja Cuesta Ruiz, Alejandro López Rico, Ana Rojo Polo, Covadonga García Grijelmo, Mikel Celorrio Reta, Eneko Martín Arroyo, Leire Artaza Aparicio, Iñaki Ituarte Aspiazu, Ane Igeregi Basabe, Itxaso Merino Julian, Isabel Diaz Rico, Maria Paz Martínez. **Hospital del Mar**: [Ramón Adalia](https://webmail.clinic.cat/owa/redir.aspx?C=pGz80-xhe0mCLX1snLoOXwbit__U9u1KVT8jGCQgYmCGotYuF2XYCA..&URL=https%3a%2f%2fpubmed.ncbi.nlm.nih.gov%2f%3fterm%3dAdalia%2bR) Bartolomé, [Luigi Zattera](https://webmail.clinic.cat/owa/redir.aspx?C=32Flt_JXDlO9ujR_eg5YxYF0ZPs25OhaEkRu2ukjfFqGotYuF2XYCA..&URL=https%3a%2f%2fpubmed.ncbi.nlm.nih.gov%2f%3fterm%3dZattera%2bL), [Irina Adalid Hernandez](https://webmail.clinic.cat/owa/redir.aspx?C=VCm4IMtwDtvV6FqIWjbdDjshvnVKXRZMg65wTxfn3tSGotYuF2XYCA..&URL=https%3a%2f%2fpubmed.ncbi.nlm.nih.gov%2f%3fterm%3dHernandez%2bIA), [Leire Larrañaga Altuna](https://webmail.clinic.cat/owa/redir.aspx?C=Ep2NK8nsaluvNSuW2WOE0v3ZsjPw6MvBPEXhVSHBlKKGotYuF2XYCA..&URL=https%3a%2f%2fpubmed.ncbi.nlm.nih.gov%2f%3fterm%3dAltuna%2bLL), [Aina Serrallonga Castells](https://webmail.clinic.cat/owa/redir.aspx?C=AL0bgUOHa5B4THljfV7SjSfgigUs-iPU0cw3WHfoV7SGotYuF2XYCA..&URL=https%3a%2f%2fpubmed.ncbi.nlm.nih.gov%2f%3fterm%3dCastells%2bAS), [Adriana Vílchez Garcia](https://webmail.clinic.cat/owa/redir.aspx?C=yS82_8bTSEt3axR2vVmlJ1yWyWwZt-p2TFfRLm-jVS-GotYuF2XYCA..&URL=https%3a%2f%2fpubmed.ncbi.nlm.nih.gov%2f%3fterm%3dGarcia%2bAV), [María Núñez](https://webmail.clinic.cat/owa/redir.aspx?C=8AJXyaxoCpsv9LYr-8v0WuWpO0UUAspfm4gXqQW8ypqGotYuF2XYCA..&URL=https%3a%2f%2fpubmed.ncbi.nlm.nih.gov%2f%3fterm%3dN%25C3%25BA%25C3%25B1ez%2bM), [Lorena Román](https://webmail.clinic.cat/owa/redir.aspx?C=Pbl7_z_mInd15pT9RoEHtmHuTqcd8RB13Jz25QT20YWGotYuF2XYCA..&URL=https%3a%2f%2fpubmed.ncbi.nlm.nih.gov%2f%3fterm%3dRom%25C3%25A1n%2bL), **Para añadir:**Isabel Ramos Delgado, Adela Benítez-Cano Martínez, Mireia Chanzá Albert, Juan Carlos Álvarez García, Luis Aguilera Cuchillo, Sandra Beltrán de Heredia, Jesús Carazo Cordobés, Carlos Alberto García Bernedo, Fernando Escolano Villén. **Hospital General Universitario De Ciudad Real:** Francisco Javier Redondo Calvo, Rubén Villazala González, Victor Baladron González, Patricia Faba, Omar Montenegro, Natalia Bejarano Ramírez. **Complejo Asistencial Universitario León:** Sergio Marcos Contreras, Alejandro Garcia Rodríguez, Saleta Rey Vázquez, Cristina Garcia Pérez, Eva Higuera Miguelez, Irene Pérez Blanco, David García Rivera. **Hospital Urduliz:** Ane Martín de la Fuente, Marta Pardo, Vanessa Rodriguez, Unai Bengoetxea. **Hospital Universitario de la Princesa:** Fernando Ramasco, Sheila Olga Santidrián Bernal, Alvar Santa Cruz Hernando, Antonio Planas Roca, Carlos Figueroa Yusta, Esther García Villabona, Carmen Vallejo Lantero, Eva Patiño Rodriguez, Alvaro Esquivel Toledo, David Arribas Méndez, Mar Orts Rodriguez, Rosa Méndez Hernández, Jesús Nieves Alonso, Inés Imaz Artazcoz, Sonia Expósito Carazo, Carlos Román Guerrero, Elena Rojo Rodríguez, Ricardo Moreno González, Julia Hernando Santos, Jara Torrente Pérez, Esperanza Mata Mena, Manuel José Muñoz Martínez, Enrique Alday Muñoz, Patricia Martin Serrano, Laura Cotter Muñoz, Amadea Mjertan, Diego Gutierrez Martínez, Carmen Rodríguez García, Olaya Alonso Viejo, Juan Alvarez Pereira, Ana Carmona Bonet, Diana Parrado López, Eva de Dios Tomas, Rafael Martín Celemin, María Luisa Meilan Paz, Luis Quecedo Gutiérrez, Noemí Diaz Velasco, Gabriel Martin Hernández, Francisco Garcia del Corral, Gloria Hernandez Arias, David Rodriguez Cuesta, Ana Gómez Rice, Encarna Mateos Sevillano, Natalia Olmos Molpeceres. **Hospital Povisa:** Beatriz Domínguez, Ana Vázquez Lima. **Hospital Ramón y Cajal:** Ángel Candela, Ismael A Acevedo Bambaren, Maria Isabel Albala Blanco, Paloma Alonso Montoiro, Fernando Álvarez Utrera, Juan Avellanosa Esteruelas, Amal Azzam López, Alberto José Balvis Balvis, Tommaso Bardi, María Beltrán Martín, Jacobo Benatar Haserfaty, Alberto Berruezo Camacho, Laura Betolaza Weimer, María del Mar Carbonell Soto, Cristina Carrasco Seral, Cristina Cerro Zaballos, Elizabeth Claros Llamas, Pilar Coleta Orduna, Ingrid P. Cortes Forero, Pascual Agustín Crespo Aliseda, María Angélica de Pablo Pajares, Yolanda Díez Remesal, Trinidad Dorado Díaz, Noemí Echevarría Blasco, María Elena Elías Martín, Javier Felices Triviño, Natalia Fernández López, Cristina Fernández Martín, Natalia Ferreiro Pozuelo, Luis Gajate Martín, Clara Gallego Santos, Diego Gil Mayo, María Gómez Rojo, Claudia González Cibrián, Elena Herrera López, Borja Hinojal Olmedillo, Berta Iglesias Gallego, Sassan Khonsari, María Nuria Mane Ruiz, María Manzanero Arroyo, Ana María Mariscal Ortega, Sara Martín Burcio, María del Carmen Martín González, Ascensión Martín Grande, Jose Juan Martín López, Cecilia Martín Rabes, Marcos Martínez Borja, Nilda Martínez Castro, Adolfo Martínez Pérez, Snejana Matcan, Cristina Medrano Viñas, Lisset Miguel Herrera, Adrián Mira Betancur, María Montiel Carbajo, Javier Moya Moradas, Lorena Muñoz Pérez, Mónica Nuñez Murias, Eva Ordiales González, Óscar Ordoñez Recio, Miguel Ángel Palomero Rodriguez, Diego Parise Roux, Lucia Pereira Torres, David Pestaña Lagunas, Juana María Pinto Corraliza, Marian Prieto Rodrigo, Inmaculada Rodriguez Diaz-Regaño, David Rodriguez Esteban, Víctor Rojas Pernia, Álvaro Ruigómez Saiz, Bárbara Saavedra Villarino, Noemí Samaranch Palero, Gloria Santos Pérez, Jaume Serna Pérez, Ana Belén Serrano Romero, Jesús Tercero López, Carlos Tiscar García, Marta de la Torre Concostrina, Eva María Ureta Mesa, Eva Velasco Olarte, Judith Villahoz Martínez, Raúl Villalaba Palacios, Gema Villanueva García, Cristina Vogel de Medeiros. **Hospital Universitario Severo Ochoa:** Soraya Gholamian Ovejero, Marta Vicente Orgaz, Patricia Lloreda Herradon, Cristina Crespo Gómez. **Hospital Universitario de Gran Canaria Dr. Negrín:** Tatiana Sarmiento-Trujillo. **Hospital de Terrassa:** Noemí García Medina, María Martínez García, Carles Espinós Ramírez, Nabil Mouhaffel Rivero, Jose Antonio Bernia Gil. **Hospital Central de la Cruz Roja San José y Santa Adela:** Sonsoles Martín. **Hospital de la Santa Creu i Sant Pau:** María Victoria Moral, Josefina Galán, Pilar Paniagua, Sergio Pérez, Albert Bainac, Ana Arias, Elsa Ramil, Jorge Escudero. **Clínica Universidad de Navarra:** Pablo Monedero, Carmen Cara, Andrea Lara, Elena Mendez Martínez, Jorge Mendoza, Íñigo Rubio Baines, Carmen Sala Trull, Pablo Montero López. Medicina Preventiva y Salud Pública: Alfredo Gea, Alejandro Montero.

**Hospital Universitario Dr. Peset Aleixandre:** Rocío Armero Ibañez, Juan Vicente Llau Pitarch, Fernando Rauer Alcóver, Cristina Álvarez Herreros, Cyntia Sánchez Martín, Lucía López Ocáriz Olmos, Marta Navas Moruno. **Complejo Hospitalario Universitario de Cáceres:** Fernando García Montoto. MF. Mirón Rodriguez, Laura Fuentes Coco, Cristina Hernández Gamito, Antonio Barba Orejudo, Luis Gerardo Smith Vielma, Yasmina González Marín, Francisco de Borja Amador Penco, Marta Donoso Domínguez, Silvia Esquivel Ramírez. H**ospital Clínico Universitario de Valencia:** José Antonio Carbonell, Berta Monleón López, Sara Martínez-Castro, Gerardo Aguilar. **Hospital Universitario a Coruña:** María Gestal, Pablo Casas, Angel Outeiro Rosato, Andrea Naveiro Pan, María Alonso Portela, Adrián García Romar, Eva Mosquera Rodríguez, Diego Ruanova Seijo, Pablo Rama Maceiras. **Complexo Universitario de Ferrol:** Francisco Castro-Ceoane, Esther Moreno López. **Hospital Clínico Universitario Lozano Blesa:** Sergio Gil, Julia Guillén Antón, Patricia García-Consuegra Tirado, Aurora Callau Calvo, Laura Forés Lisbona, María Carbonell Romero, Belén Albericio Gil, Laura Pradal Jarne, María Soria Lozano, Diego Loscos López, Andrea Patiño Abarca. **Universal Doctors:** Jordi Serrano. **UBIKARE**: Javier Pérez-Asenjo, Ángel Díez-Domínguez, Ion Zubizarreta, Jon Ramos, Iosu Fernández. **Hospital Universitario La Paz:** Emilio Maseda, Alejandro Suárez de la Rica, Javier Veganzones, Itziar Insausti, Javier Sagra, Sofía Díaz Carrasco, Ana Montero Feijoo, Julio Yagüe. **Hospital Universitario Gregorio Marañon:** Ignacio Garutti. **Hospital San Joan Despí Moises Broggi:** Eva Bassas Parga, Carmen Deiros Garcia, Elisenda Pujol Rosa, Ana Tejedor Navarro, Roser Font Gabernet, Maria José Bernat, Meritxell Serra Valls, Cristina Cobaleda Garcia-Bernalt, Jesus Fernanz Anton, Adriana Aponte Sierra, Lucia Gil Gomez, Olaia Guenaga Vaqueiro, Susana Hernandez Marin, Laura Pardo Pinzon, Sira Garcia Aranda, Carlos Briones Orejuela, Edgar Cortes Sánchez, Alejandro Romero Fernández, Esther Fernández SanJosé, Patricia Iglesias Garsabal, Guillermo Isidro Lopez, Ana Vicol, Sara Espejo Malagon, María Sanabra Loewe, Laura Grau Torradeflo, Lourdes Blanco Alcaide, Gloria Buenaventura Sanclemente, Pere Serra Pujol, Gustavo Cuadros Mendoza, Miroslawa Konarska, Fedra Bachs Almenara, Agnieszka Golska, Aleix Carmona Blesa, Arantxa Mas Serra. **Hospital Universitario Infanta Leonor:** Javier Ripolles Melchor, Ana Nieto Moreno, Káteri Chao Novo, Sandra Gadín López, Elena Nieto Moreno, Bérénice Gutiérrez Tonal, Elena Lucena de Pablo, Barbara Algar Yañez, Beatriz Vázquez Rivero, Beatriz Nozal Mateo, Marina de Retes, Norma Aracil Escoda, Cristina Gallardo Mayo, Rosa Sanz González, Alicia Ruiz Escobar, Maria Laura Pelegrina López, Marina Valenzuela Peña, David Stolle Dueñas, Ane Abad Motos, Alfredo Abad-Gurumeta, Ana Tirado Errazquin, Elena Sáez Ruiz, Nerea Gómez Pérez, Francisco de Borja Bau González. **Hospital sanitas CIMA:** Cesar Morcillo Serra, Jessica Souto Higueras. **Hospital Universitario y Politécnico La Fé:** Rosario Vicente, Raquel Ferrandis, Silvia Polo Martín, Azucena Pajares Moncho, Ignacio Moreno Puigdollers, Juan Pérez Artacho Cortés, Ana Moret Calvo, Ana Pi Peña, María Catalán Fernández.

**Complexo hospitalario Universitario de Pontevedra:** Marina Varela, Pilar Díaz Parada, Raquel Rey Carlín, Sarra Barreiro Aragunde. **Hospital Arnau de Vilanova**: María Isabel Forés Chiva. **Hospital General de Alicante:** A. Javier Agulló. **Hospital Universitario Infanta Sofía:** Antonio Pérez Ferrer. **Hospital Universitario San Juan de Alicante**: María Galiana. **Hospital Nuestra Señora de Meritxell SAAS:** Antoni Margarit, Válerie Mourre del Rio, Eva Heras Muxella, Anna Vidal.
